# Community-engaged design of a virtual diabetes prevention program intervention with care coordination

**DOI:** 10.1186/s12913-026-14860-0

**Published:** 2026-06-13

**Authors:** Katherine LaMonaca, Meredith Campbell-Britton, Kathleen O’Connor Duffany, Beth P. Comerford, Sofia I. Morales, Rockiy G. Ayettey, Katia Astudillo, Mary Swansiger, Claudette Kidd, Bailee Rue, Alycia Santilli, Valentine Y. Njike, Todd McGuire, Maryann Deyling, Rafael Pérez-Escamilla

**Affiliations:** 1 Yale School of Public Health, 135 College Street, Suite 200, New Haven, CT 06510 USA; 2 Yale School of Medicine, 333 Cedar Street, New Haven, CT 06510 USA; 3 Griffin Hospital, 130 Division Street, Second Floor, Derby, CT 06418 USA; 4 Project Access New Haven, 63 York St, New Haven, CT 06511 USA; 5 College of Health and Human Services, Southern Connecticut State University, 493 Fitch St, New Haven, CT 06514 USA; 6 IncentaHEALTH, LLC, 9605 S. Kingston Court, Suite 130, Englewood, CO 80112 USA

**Keywords:** Type 2 Diabetes, Prediabetes, Prevention, Telemedicine

## Abstract

**Background:**

The Diabetes Prevention Program (DPP) is an evidence-based approach to prevent Type 2 Diabetes (T2D), the leading cause of morbidity and mortality in the United States. Despite demonstrated benefits, acceptability and uptake of DPPs remain low, especially among communities with low incomes at increased risk for diabetes. The Yale-Griffin Prevention Research Center led the design of a community-engaged virtual DPP (vDPP) intervention and assessed its feasibility of implementation in two contrasting settings.

**Methods:**

The vDPP design followed an iterative, multi-partner mixed methods approach guided by a community*-*engaged research framework. The steps involved were: (1) application of the socio-ecological model theoretical framework to vDPP design (2) interviews and a technology survey with community residents and providers to assess feasibility (3) development of a program impact pathway (PIP) for ongoing process evaluation (4) five-week pilot study followed by participant focus groups, and (5) triage of findings to make necessary adaptations.

**Results:**

This process led to a vDPP intervention that included an online platform with asynchronous programming and a health coach as well as an added care coordination component provided by local community health workers and hospital-based community nurses. Thematic analysis showed that community members and providers valued the concept of a structured program with personalized guidance and emphasized the importance of addressing social needs and considering broad health goals. The pilot study identified program facilitators and barriers around engagement, communication, and feelings of personal accountability that informed program adaptations made prior to full implementation testing, including clarification of support roles and addition of detailed orientation materials.

**Conclusions:**

This formative research demonstrated initial program feasibility and identified ways to improve the quality of program delivery. Pairing the PIP with a community-engaged approach led to the development of a novel health promotion intervention that is now being tested through a subsequent implementation science study.

**Trial registration:**

NCT04195477, clinicaltrials.gov, registered 7 October, 2020; https://www.clinicaltrials.gov/study/NCT04195477?term=NCT04195477&rank=1.

**Supplementary Information:**

The online version contains supplementary material available at 10.1186/s12913-026-14860-0.

Contributions to the literature • The CDC’s Diabetes Prevention Program (DPP), an evidenced based intervention to prevent and delay the onset of Type 2 diabetes, is underutilized nationwide, especially in communities with low incomes.

• More research is needed on how communities with low incomes could benefit from a virtual Diabetes Prevention Program (vDPP).

• A community-engaged formative research approach coupled with a detailed quality improvement analysis helped design and adapt a virtual diabetes prevention program (vDPP) with strong potential for robust acceptability and uptake among two contrasting communities with low incomes.

## Background

Type 2 diabetes (T2D) is a leading cause of morbidity in the United States, affecting 11% of the population [1]. Another 38% of adults are estimated to have prediabetes, characterized by heightened blood sugar that may lead to T2D [2]. Diabetes contributes to significant morbidity and mortality and leads to exceedingly high medical expenditures [1, 3]. Across Connecticut, an estimated 367,000, or nearly 13% of adults, have Types 1 or 2 diabetes, a quarter of whom are likely to be undiagnosed [4]. The statewide prevalence is higher among those with lower household incomes and educational attainment, and there are significant inequities by race and ethnicity, with a higher prevalence among non-Hispanic Black (15.5%), Hispanic (13.8%), and non-Hispanic other populations (10.1%) compared to white populations (7.0%) [4]. 

The National Diabetes Prevention Program (DPP) is a CDC-approved, comprehensive lifestyle intervention program developed in 2010 to prevent or delay the onset of T2D among at-risk adults. To address known risk factors for T2D, the program emphasizes healthy eating, physical activity, and stress management [5]. CDC-certified DPPs follow a yearlong curriculum that includes one-on-one support from trained lifestyle coaches [6]. DPPs are traditionally delivered with in-person workshops and coaching support, but there is a rising trend toward remote and hybrid formats [7]. The original DPP demonstrated a positive, sustained effect on reducing diabetes incidence [8, 9]. The DPP has since been successfully translated to a variety of real world settings and has been shown to be a more cost-effective alternative than medication-based interventions [10]. 

Despite these benefits, research shows that DPPs remain substantially underutilized nationwide [11]. There are multiple challenges associated with sustained engagement in lifestyle prevention programs, especially among populations with lower incomes at increased risk for T2D. Documented barriers to engagement include the cost of programs to participants, limited access to healthy foods, limited time for cooking and exercising, limited access to safe physical spaces for exercise, and cognitive or physical disabilities [12]. Additional challenges associated with in-person lifestyle prevention programs include transportation, safety concerns, accessibility, and availability of child/elder care [13, 14]. Multiple studies have called for innovative partnerships and culturally congruent communication with program participants, as well as further evaluation of implementation outcomes [15–18]. 

Research suggests several potential ways that DPP delivery can be modified or supplemented to address these documented barriers. Virtual delivery of DPPs (vDPP) is a promising and increasingly attractive strategy to increase engagement by overcoming time and logistical constraints and potentially reducing costs [19, 20]. Two pilot studies found virtual, asynchronous delivery to be effective in terms of health outcomes and cost [21–23]. Along with these strategies, some programs have enhanced engagement by incorporating personal coaching into their delivery models [24, 25]. Other possible supplemental program components may also help to mitigate barriers related to knowledge and access, such as community health workers that can improve linkages to community resources and social services.

**Fig. 1:**
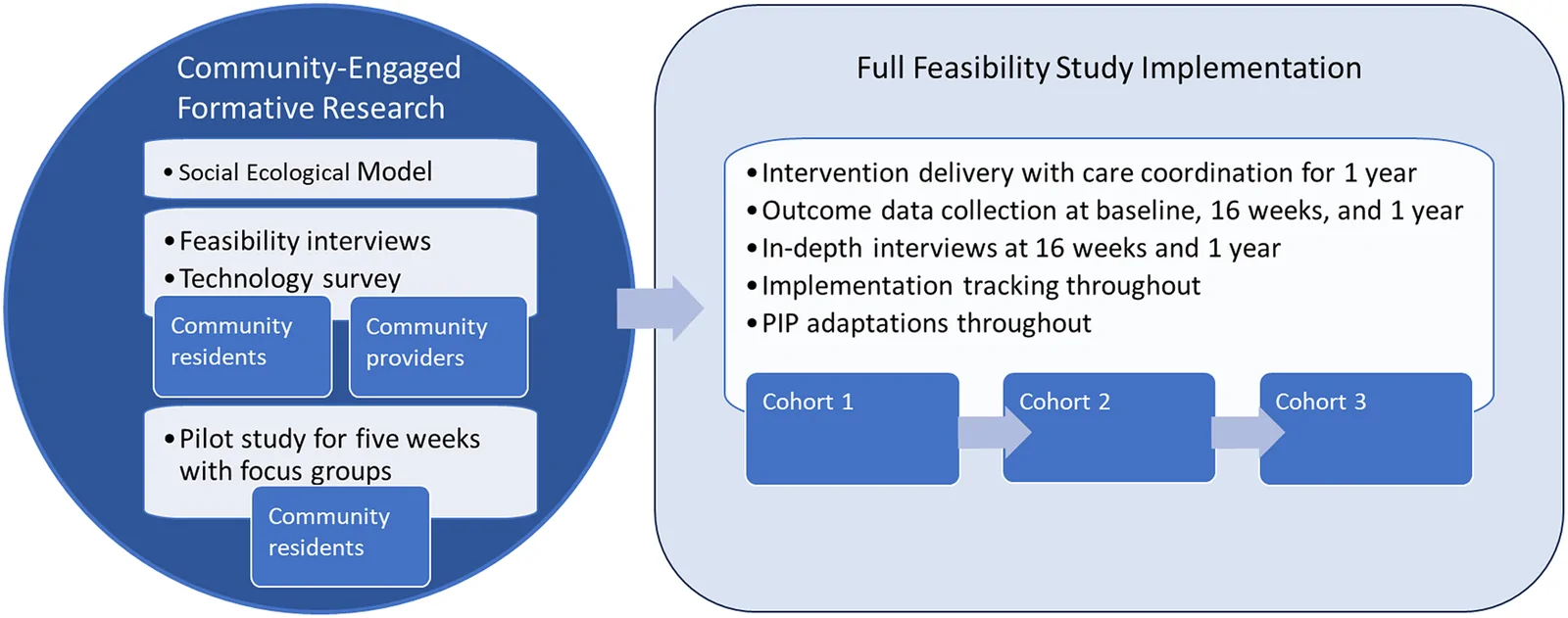
Timeline of virtual Diabetes Prevention Program implementation feasibility study. This article focuses on the formative research phase. Abbreviation: PIP, Program Impact Pathway

With promising research demonstrating the initial health benefits of vDPPs in communities with low incomes, more data is needed on how to effectively deliver this intervention in the context of competing health and social needs [26]. To address this gap, the Yale-Griffin Prevention Research Center (Y-G PRC) applied a community-engaged approach to design a vDPP intervention and conduct a feasibility study in diverse communities (see Fig. 1 for study timeline). In this paper, we describe the formative research process, guided by a program impact pathway (PIP) quality improvement analysis, and our adapted vDPP intervention.

## Methods

**Fig. 2:**
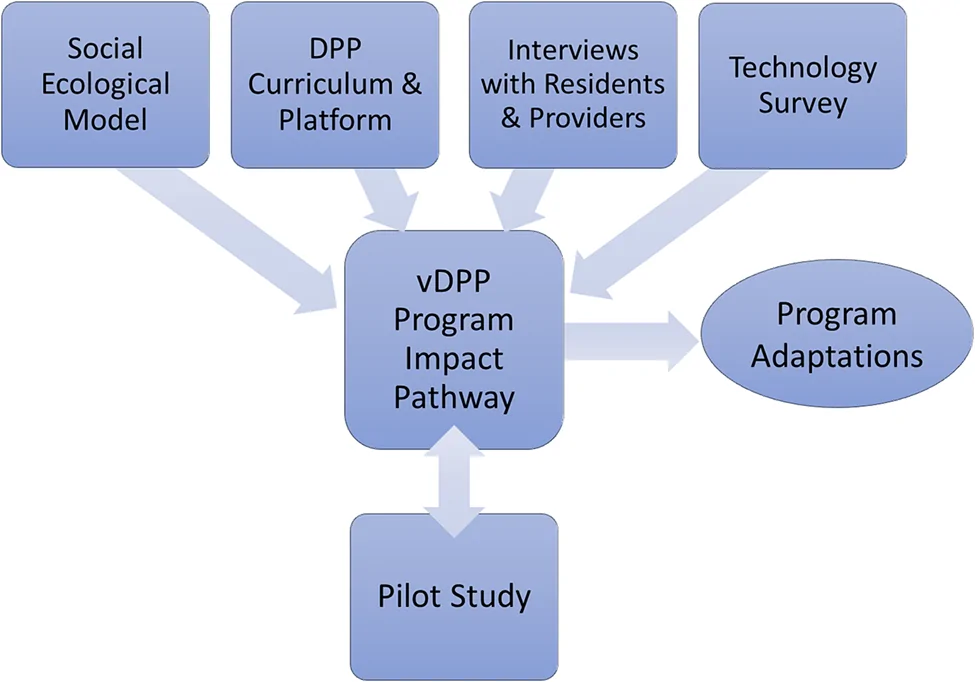
Methods for co-design of virtual Diabetes Prevention Program implementation study. Abbreviations: DPP, Diabetes Prevention Program; vDPP, virtual Diabetes Prevention Program

Our formative vDPP intervention community-engaged design process included five main steps: [1] application of the socio-ecological model to the standard DPP program and curriculum to identify potential barriers and facilitators for implementation in communities with low incomes [2] interviews and a technology survey with community residents and providers to assess program acceptability and identify potential barriers [3], development of an initial study PIP; [4] a five-week pilot intervention with post-program focus groups to test implementation, and [5] integration of pilot results with PIP critical quality control points to make program adaptations. Steps 1 and 2 informed the design of the PIP, while Step 4 (pilot study) was guided by the PIP and informed Step 5 (program adaptations) *(*see Fig. 2*)*. This iterative, multi-partner, and mixed methods approach allowed the research team to develop a study intervention that was responsive to health and social needs. This manuscript adheres to reporting guidelines for qualitative research and for intervention design [27, 28]. See Additional Files 1 and 2 for completed checklists.

### Theoretical framework

We used the socio-ecological model as the guiding framework for the initial vDPP implementation program design, aiming to address identified barriers to lifestyle change programs previously reported by our communities. These included logistical matters such as lack of time and transportation, as well as more systematic barriers like lack of access to healthy, fresh, and preferred foods [29, 30]. The model includes an overarching set of theoretical principles to understand the interrelations among diverse personal and environmental factors in health and illness [31]. It has been used to explain systems change in health promotion programming by illustrating the interactions between individuals and systems that impact behavior and health in diverse settings [32–39]. 

We hypothesized that the vDPP would be most effective if designed to intervene at multiple levels of influence, including individual (vDPP curriculum delivery), interpersonal (care coordination and social support), organizational (standards of practice for hospital and community organizations), and community (environmental changes). We identified a need for asynchronous curriculum delivery to address barriers such as transportation, childcare, and other responsibilities. A selection team, including two community research consultants, a community health worker (CHW), and a hospital-based community health nurse, met regularly over six months to discuss and select a vDPP platform for program use. Community research consultants were local residents trained on health inequities who collaborated with study investigators. They received formal training in research practices and contributed to study design, development, and operations. This team deliberated on several criteria aligned with literature review findings and the socio-ecological model, including [1] method of core vDPP curriculum delivery (asynchronous vs. synchronous, frequency) [2], role of the health coach [3], type of support group features, and [4] physical activity/meal tracking approaches.

### Interviews

To assess initial program feasibility and acceptability, we conducted semi-structured interviews with community residents (*n* = 21) and health and social service providers (*n* = 13) living or working in our communities of focus within the Lower Naugatuck Valley and New Haven, Connecticut. All interviews were conducted in English. Participation in the interviews was voluntary and did not affect respondents’ relationships with study partners. Community residents received a $25 gift card in appreciation for their time.

All interviews were conducted online due to COVID-19 pandemic restrictions (June-November 2020). They were scheduled at respondents’ convenience and ranged from 16 to 56 min in length. The data were recorded, professionally transcribed, de-identified, and managed through qualitative software (QSR International Pty Ltd. Version 12, 2018). Interviewers wrote field notes to capture salient details and experiences. Analysts co-developed separate codebooks to manage data from each set of interviews. Team members developed an initial list of codes from independent reviews of three to four transcripts. One team member consolidated the codes for peer review. Smaller teams then applied the codes to the remaining transcripts, developing preliminary findings that were later refined using the RE-AIM analytical framework and PIP evaluation tool.

Researchers reviewed findings in progress with the full research team, study partners, and the Y-G PRC Community Advisory Group (CAG) for credibility [40]. The CAG is a diverse group of residents who live in the Y-G PRC communities of focus. They review and provide input on all aspects of the Y-G PRC’s community-based research.

### Community resident interviews

We used convenience sampling to recruit interview respondents from our communities of focus. We posted flyers at various sites within the study communities and emailed flyers via listservs from study partners and community-based organizations. Flyers were designed with input from study partners, community research consultants, and a hospital-based community nurse. Interested residents were screened by phone for eligibility according to the following criteria [1] over 18 years of age [2] resident of a public housing property (New Haven) or a regional town (Lower Naugatuck Valley) [3] no previous diagnosis of T2D [4], at risk for diabetes according to the CDC diabetes risk screen [5, 41] household income at or below state criteria for Medicaid eligibility. Approximately 90% of respondents (*n* = 19) met the CDC criteria for pre-diabetes and 10% (*n* = 2) were diagnosed with T2D by a clinician.

The resident interview guide was developed collaboratively by the Y-G PRC research team and community research consultants to identify individual and community needs, barriers, and resources that could affect intervention feasibility (see Additional File #3).

### Provider interviews

Researchers used a purposive sampling strategy to recruit health and social service providers working in the communities of focus. They emailed professional contacts to invite participation and to request recommendations for potential participants (see Additional File #4 for sample characteristics). The provider interview guide was designed to elicit feedback on community needs and program feasibility and acceptability, including issues that might affect participant enrollment and engagement (see Additional File #3). The guide was developed collaboratively by the Y-G PRC research team and reviewed by a study partner.

### Technology survey

We administered a brief survey to community resident interview participants to assess information technology access and comfort, a component of overall program acceptability and feasibility. The Qualtrics survey included quantitative items on location and frequency of internet access, comfort with accessing the internet, and experience using internet devices (computer or smartphone) to monitor diet and/or exercise (see Additional File #5). Nineteen of the 21 invited community residents completed the survey.

### Quality improvement approach

After identifying a vDPP platform and conducting the feasibility interviews and technology survey, we used the Program Impact Pathway (PIP) quality improvement approach to detail expectations and monitor processes for how the vDPP would achieve anticipated impacts [42, 43]. A PIP diagram can be considered a “roadmap” that program participants would need to follow to receive the intended program benefits. It builds on the concept of logic models to support implementation fidelity and program impact. A PIP framework facilitates the identification and monitoring of critical quality control points (CCPs) to ensure that all program activities and processes are functioning to achieve the intended program outcomes. It serves as a tool to identify potential bottlenecks or issues that may be impacting the program’s progress and make necessary adjustments to improve effectiveness.

We developed the initial study PIP based on the DPP CDC curriculum and selected vDPP online platform. We started with a logic model and mapped out each step in program recruitment, enrollment, and implementation along with short, medium, and long-term outcomes (see Additional File 6 for logic model). The PIP visually demonstrates the relationships between the vDPP program components, intended outcomes, and any contextual factors that may impact those outcomes.

### Pilot study

Following the program design, feasibility interviews and technology survey, and initial PIP analysis, we conducted a five-week pilot study to test key implementation activities, including onboarding and orientation of program clients. We planned for the pilot to identify potential challenges and solutions prior to launching the full feasibility study to be published separately. In May 2021, we conducted recruitment by first contacting previous community resident interview participants, followed by outreach through public housing authorities. We screened participants according to the same inclusion criteria applied for community resident interviews along with a willingness to enroll in the study for five weeks. Potential participants were excluded if they were pregnant or planning to become pregnant, if they reported a current eating disorder or being in recovery for an eating disorder, if they were directed by a physician not to engage in physical activity, or if they reported any other circumstances that would prevent them from completing the program. Twenty-five individuals from New Haven (*n* = 12) and the Lower Naugatuck Valley (*n* = 13) were screened for eligibility. Thirteen people met pilot eligibility criteria, and 11 people were enrolled (5 from New Haven, 6 from the Lower Naugatuck Valley). Two eligible people declined enrollment - one due to lack of access to a smartphone and the other due to time commitment. Demographic characteristics of pilot participants are described in Table 1.

**Table 1:** Demographic characteristics of participants enrolled in the pilot study of the virtual Diabetes Prevention Program

Characteristic	All Participants (*N* = 11)	Lower Naugatuck Valley (*n* = 6)	New Haven (*n* = 5)
Age (median, range)	54.5 (34–60)	46.0 (34–60)	56.0 (34–57)
Gender			
Male	1 (9.1)	1 (16.7)	0 (0.0)
Female	10 (90.9)	5 (83.3)	5 (100.0)
Ethnicity			
Hispanic	1 (9.1)	1 (16.7)	0 (0.00)
Non-Hispanic	10 (90.9)	5 (83.3)	5 (100.0)
Race			
Black / African-American	4 (36.4)	0 (0.0)	4 (80.0)
White	7 (63.6)	6 (100.0)	1 (20.0)
Marital Status			
Divorced	1(9.1)	0 (0.0)	1 (20.0)
Married	4	4 (66.7)	0 (0.0)
Never Married	5 (45.5)	1 (16.7)	4 (80.0)
Widowed	1 (9.1)	1 (16.7)	0 (0.0)
Education			
Associates Degree / Some College	3 (27.3)	0 (0.0)	3 (60.0)
Graduate Degree	2 (18.2)	2 (33.3)	0 (0.0)
High School or Less	6 (54.5)	4 (66.7)	2 (40.0)
Employment Status			
Disability	5 (45.5)	3 (50.0)	2 (40.0)
Employed Full Time	2 (18.2)	2 (33.3)	0 (0.0)
Self-Employed	1 (9.1)	0 (0.00)	1 (20.0)
Unemployed	3 (27.3)	1 (16.7)	2 (40.0)
Insurance Status			
Medicaid	9 (81.8)	4 (66.7)	5 (100.0)
Medicare	2 (18.2)	2 (33.3)	0 (0.0)

^1^All variable frequencies are reported as *n* (%) except Age, as otherwise noted

^2^ Percentages may not equal 100 due to rounding

Pilot participants (*n* = 11) were followed for five weeks, with the option to continue on the platform for a full year outside of the study. The vDPP intervention included enrollment on the selected platform, access to the 26 DPP curriculum webinars, the platform’s digital smart scale for weekly weigh-ins, access to the platform’s health coach, and regular contact with a CHW (New Haven) or a hospital-based community nurse (Lower Naugatuck Valley). We also provided a wearable activity tracker that could sync to the platform for additional tracking support.

We assessed the pilot study results using the RE-AIM evaluation framework in combination with the PIP process to make needed program adjustments and to test different feasibility assessment methods during the full intervention implementation in a subsequent study. The RE-AIM framework includes measures of reach, effectiveness, adoption, implementation, and maintenance [44]. For program reach, we assessed the number of participants screened, eligible, and enrolled in the pilot program. In the pilot, we tested measures of program effectiveness by capturing participant behavior and knowledge using a baseline and follow-up survey that included standardized instruments for dietary intake [45], physical activity levels [46], diabetes risk knowledge [47], nutrition self-efficacy [48], food security [49], and perceived stress [50]. While we gathered these data to assess instrument questions, we do not report them here as we did not anticipate significant changes during the five-week duration of the pilot. For the same reason, we did not measure biometric outcomes in the pilot test, such as weight, BMI, and HbA1c. We used an adapted version of the Accountable Health Communities Health-related Social Needs Screening Tool at program baseline to identify social determinants of health needs for which the CHW/HCN would provide resources and navigation support (see Additional File #7) [51]. 

For adoption, we collected data from the vDPP platform to measure participant program engagement, including number of webinars watched, weigh-ins completed, and physical activities logged. We also measured participant satisfaction through a structured survey addressing program experience, program engagement, and time required to complete each program component (see Additional File #8 for participant satisfaction survey) [52]. For implementation, the study team kept an implementation tracker to document issues and resolutions, segmenting issues by PIP CCPs. Due to the nature of a pilot program, we did not assess measures of program maintenance.

Participants were invited to take part in focus groups after pilot completion to describe their experiences throughout the program. Nine of 11 pilot participants participated in two focus groups. The discussion guide was developed collaboratively by researchers and reviewed for credibility by the Y-G PRC CAG [40]. The focus groups were co-facilitated by a social work researcher, a public health researcher, and a community research consultant. Sessions were conducted online, recorded, and professionally transcribed. Participation was voluntary and did not affect participants’ relationships with any study partners. Transcripts were de-identified, verified for accuracy, and augmented with field notes. The analytic team used a structured template to identify facilitators and barriers to engagement, experiences with program components, and recommendations for improvement [53, 54]. These programmatic findings were critically reviewed with the larger research team and the Y-G PRC CAG to determine and confirm adaptations.

## Results

### Intervention community-engaged design

#### Setting & partners

To design a meaningful intervention that could act upon multiple levels of influence, the Y-G PRC partnered with representatives from social service, health, and community research organizations serving cities and towns with a high prevalence of chronic disease. Collaboration with a variety of community partners was important to facilitate the translation of study findings to potentially influence systems change. In New Haven, we partnered with the Community Alliance for Research & Engagement, who then facilitated key partnerships with a local housing authority and a community-based organization specializing in CHW services. In the Lower Naugatuck Valley, the main partner was Griffin Hospital, a community-based hospital.

Also as part of our theoretical approach using the social-ecological model, we identified that having a specific, geographically defined scope for each study site would allow the study team to potentially recognize and inform needed community, systems, and policy changes related to social determinants of health, through the identification of common participant barriers. Collectively, our research team and partners identified two communities of focus within the Y-G PRC catchment area:

- New Haven is one of the largest, most diverse cities in Connecticut, with 72% of residents identifying as people of color. Nearly half of residents (49%) are considered cost-burdened, spending at least 30% of their income on housing. They also report higher rates of smoking, obesity, and food insecurity and lower rates of exercise compared with statewide averages [55]. Study partners determined that eligibility criteria for New Haven residents would include living in a designated housing authority location, with the housing authority supporting recruitment.
- In the two suburban towns in the Lower Naugatuck Valley, 38% to 44% of residents identified as people of color. Both towns reported higher rates of cost burden, smoking, and obesity and lower rates of exercise compared with statewide averages. The rate of food insecurity in the Lower Naugatuck Valley is comparable with state averages [56, 57]. Hospital study partners agreed to support recruitment through their social service and health programs.

#### vDPP delivery

The study platform selection team considered 15 potential vDPP delivery platforms during the review process. The intervention platform, IncentaHEALTH, was chosen due to its availability in multiple formats (mobile application or web browser), its asynchronous design that allowed flexibility in completing program components, the availability of trained health coaches to provide one-on-one support to participants and work with CHWs/hospital-based community nurses, and built-in tracking tools for weight and physical activity. From a research study perspective, the selection team valued that the platform provided detailed data collection functionality on participant engagement.

The main DPP components of the IncentaHEALTH online platform included [1] 26 asynchronous educational webinars on DPP Preventing T2D curriculum content [2], function for completing weekly weigh-ins using a provided smart scale along with a “healthy selfie” photo to track visual progress, and [3] ability to log a variety of physical activities on a weekly basis. This content is aligned with the DPP curriculum and designed to last for one year, with webinars delivered weekly for the first 16 weeks and the remaining 10 webinars delivered over the final 8 months. The platform also provided optional supplementary components, including weekly meal plans with recipes and shopping lists, a dietary intake tracker, exercise plans and videos, guided meditations, and a monetary weight loss incentive component, to be distributed quarterly based on percentage of body weight lost. In addition to platform access, platform health coaches conducted regular check-ins via email, text, or phone every one to four weeks depending on the program phase.

All IncentaHEALTH platform content was originally developed at a 6th grade reading level on the Flesch-Kincaid Grade Level scale. The 26 sessions were developed by incentaHEALTH Diabetes Prevention Program Health Coaches, who were trained in the national Diabetes Prevention Program Lifestyle Coaching program, and who also are Certified Personal Trainers and trained in motivational interviewing. Each of the 26 sessions contained prompts and pause breaks where participants were prompted to submit questions to the “Health Chat” feature in the platform to interact with other participants and DPP Health Coaches. The sessions also regularly prompted the participant to reach out to the DPP Health Coach to get additional support with the program.

#### Care coordination

To supplement the online program and address potential barriers related to social determinants of health, we designed the intervention to include a local care coordination component for all study participants. In New Haven, we planned for participants to receive support from a trained CHW affiliated with a study partner organization with extensive knowledge of local resources. A hospital-based community nurse (HCN), also well-versed in local services, would provide the same support to participants in the Lower Naugatuck Valley. To identify potential social determinants of health, CHWs/HCNs would administer a health-related social needs screening questionnaire at the start of the program and conduct follow-up check-ins by phone every one to two weeks to coordinate access to local health and social resources, such as food pantries, utilities and rental assistance, and connections to primary care, and troubleshoot any issues with the platform.

#### Feasibility and acceptability

Interview respondents (community residents and providers) generally endorsed the feasibility and acceptability of this vDPP intervention (see illustrative quotes in Table 2). Most respondents felt that people would welcome a program like this, seeing it as a chance to act early on a serious health issue and possibly break personal or family histories of chronic illness. They thought that the structured program and formal support could help people initiate and sustain positive health behaviors. Resident respondents were interested in receiving direct, personalized guidance around cooking, meal planning, and exercise, and all respondents highlighted the importance of the program’s flexible scheduling in removing participation barriers.

**Table 2:** Community resident and provider interview quotes regarding feasibility and acceptability of virtual Diabetes Prevention Program

Themes	Illustrative Quotes
Interest in program	“I have COPD, I have high blood pressure, which runs in my family. I lost both my parents to cancer, which is very scary to me…I don’t have diabetes now, but I want to try to eat and stay as healthy as I could because I don’t want any more problems (R-12).”
	“I think a lot of residents have been told they’re pre-diabetic and they’re worried” (P-05)
Direct, personalized guidance	“I remember portion of my son’s life while he enjoy a gym, he had an instructor…. Because it’s better to have someone that knows what we doing, what we saying because it will make it better, will make it more professional for us, more worth it.” (R-04)
	“Everything isn’t for everybody….So what works for one person doesn’t always work for another person, but it wouldn’t [hurt] to try it and see what the results are.” (R-07)
	“[My doctor] says, “You need to lose some weight. If you lose the weight you’ll feel much better,” but I’ve been trying everything, so maybe I need a regimen where somebody could tell me what to eat, break down my portions, and do that.” (R-05)
Other health goals	“I can be healthy eating, but if my mind is crazy it’s not going to happen, it isn’t going to help me anyway. It has to be a balance of the three. Exercising, eating, and mind” (R-19)
	“I think I’m someone who tends to focus on mental and emotional health first; that’s why it’s difficult to focus on the physical aspects of health, such as diet and weight-loss, when I’m under a lot of stress and depression.” (R-20)
	“being able to just live normal, not have to worry about having any health issues. Just basically eating the right proportion to food and just taking care of yourself, little exercise and everything” (R-22)
	“to be able to have a healthy body, to have the energy, the self-esteem. To be able to feel good about yourself and overall general health” (R-16)
	“healthy to me means I can live longer” (R-13)
Technology comfort and support	“But now because of how it is and how the projection is it’s going to continue to stay with us for longer, I think our residents, even the ones who don’t want anything to do with technology are understanding. I mean, your medical appointments are all done this way….I think that they’re buying into it more just out of the necessity to do it.” (P-16)
	“things that I never would have thought, like when we’re enrolling patients the kind of phone, no memory, no space, no knowing password. So many issues.” (P-03)
Peer support	“I think talking to others, sharing stories with others, the positives, maybe the negatives, I think it would help out a lot. Because we could help each other out too, to get through the hard times.” (R-16)
	“I think if they really do need that in-person touch, the socialization connection, I’m not sure if the online would give them enough.” (P-12)
	“-If we have a good group, we let them kind of partner and exchanged phone numbers, and they were calling each other. So they were reminding that on the action plans and the next meeting. […] So just for me to call them it makes such a difference that they were just looking for the next meeting to be able to be in a group and being able to talk. Talk about he way they feel, talking about their accomplishments, talk about how they were managing their diet and making changes. They were happy because they have the opportunity.” (P-07)
	“Okay, you made a mistake. That’s all right, brush yourself off, get back up and get back on the wagon and try it again….That’s a part of getting free of what you’re doing. It puts you on the path of okay, I’m not going to give up. Okay, I had a setback. That’s all right, I’ll just get back up and do it again and just take that. If I gained a pound you’ve got to work that pound off again.” (R-13)
	“Over time it created a sense of community because they see the same people each week and they’re able to relate. I think that was huge, it was like oh, I’m going to wellness program but I made friends there and I’m coming because not only am I learning about wellness and nutrition and cooking, but I’m also going to be there with my friend that I’ve made. So I think that’s important.” (P-09)
	“Sometimes I can get absorbed into feeling that I am the only person that’s going through this….So I think talking to others, sharing stories with others, the positives, maybe the negatives, I think it would help out a lot. Because we could help each other out too, to get through the hard times.” (R-16)
Program support	“I’ve seen too many coaches just didn’t care. ‘Well, I can do this already, it’s up to you.’ You don’t say that to a person who’s trying to lose weight or trying to stay healthy or changing my lifestyle to be healthy (R-09).”
	“’I’m just calling to see if you’re all right. And if there’s anything that I can help with.’ I’m not calling to say you haven’t logged in and where are you? Why aren’t you doing this? I’m not judging you, just that friendly phone call.” (P-18)
	“being on teleconference, they weren’t feeling the support that they felt in person. And some of them did drift off and I have not heard from them. I know they’re healthy, they’ve told me they’re okay. But they have not come back to the group (P-12).”
	“It could be that they can’t read the paper. They can’t do this step as easily as you all think they can. Or they need the reminder. They got the date wrong and they missed the meeting, so now they’re frustrated because they missed one meeting and they think they’re going to be scolded so they’re not going to come to the next meeting because they dismissed the wrong information or whatever the case may be. It’s a lot of hand-holding, it’s a lot of reminding, and it’s a lot of encouragement.” (P-05)
	“Just having checks and balances, some types of quality assurance…then you can also be prepared to recalibrate a little so that you don’t lose people.” (P-10)
Increasing social needs in community	“someone had asked, ‘Where can I find children’s clothes, used, donated children’s clothes.’ Those are really big needs I did not see last year.” (P-09)
	“people are just trying to get by and figure out, ‘How do I get through the next day or the next week? How do I keep the lights on?” (P-10)”
	“it’s hard when I only get a certain amount of food stamps and it’s me and my son, so I have to go low and cheap” (P-01)

^1^All interviews were conducted in 2020 and findings may be affected by health and social impacts of the COVID-19 pandemic

Some respondents felt that community residents may not consider diabetes prevention to be a priority, whether due to their health beliefs or their interest in other health goals. They also identified significant social needs, like caregiving and financial hardship, that they felt were worsened by the COVID-19 pandemic. They emphasized that these stressors could derail people’s efforts to prioritize their health.

To support program engagement, respondents recommended tailoring the intervention to be inclusive of other meaningful health outcomes, like losing weight, reducing stress, or taking fewer medications. These adjustments reflected the broad vision of physical, mental, emotional, and financial health that community residents described during the interviews. Respondents suggested incorporating technical support to help build digital skills and peer support with optional in-person activities to offset isolation or loneliness associated with COVID-19 social distancing guidelines. Finally, respondents advocated for the program team to conduct frequent check-ins to get to know participants, to see how they are doing outside of the program, and to understand their experiences with the intervention.

The technology survey results were favorable towards the use and acceptance of an online platform. While many survey respondents reported that they had not previously used a computer or smartphone to track their physical activity or dietary intake (*n* = 13, 68.4%), most had access to wireless networks and devices at home (*n* = 16, 73.7%) and were comfortable navigating online sites and applications independently (*n* = 16, 73.7%). Detailed technology survey results are available in Additional File #9.

#### Program impact pathway quality improvement approach

**Fig. 3:**
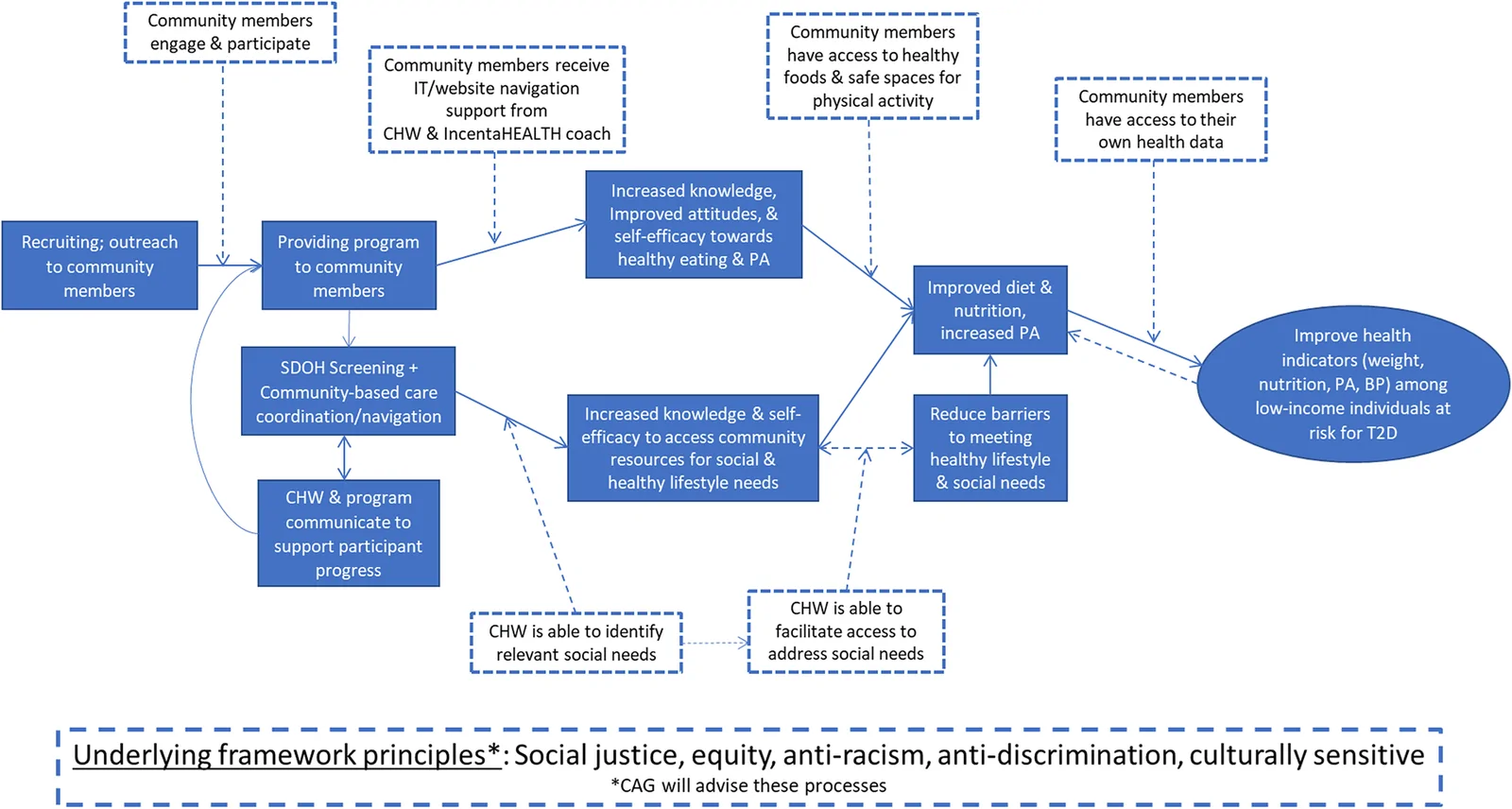
Initial program impact pathway for quality improvement of the virtual Diabetes Prevention Program intervention. Key program activities are represented by solid blue boxes, arrows show the direction of activities, critical quality control points are represented by boxes with intermittent lines, and the desired impact of the program is shown in an oval at the end of the diagram. Abbreviations: CAG, Community Advisory Group, CHW, Community Health Worker

We created the initial PIP (see Fig. 3) with operational CCPs that reflected the monitoring needs of the program components and desired intervention outcomes. These CCPs enabled the research team to identify metrics to monitor key program processes, barriers, facilitators, and outcomes in a systematic and timely way (see Table 3).

**Table 3:** Assessment plan for critical quality control points in virtual Diabetes Prevention Program intervention in two low-income communities in Connecticut

Critical Quality Control Point (CCP)	Assessment Tools	Metrics	Context
Community members engage and participate	• Pilot enrollment data • Intervention enrollment data • Platform engagement data • Implementation tracker • Focus groups with pilot participants • Follow-up interviews with full intervention participants • Satisfaction survey	• # screened • # eligible • # enrolled • #/% webinars watched • #/% weigh-ins completed • #/% physical activity logged • Δ satisfaction survey	• Participant reports of facilitators and barriers
Community members receive information technology and/or website support from CHW/HCN^1^ and health coach	• Technology survey • Implementation tracker • Pilot focus groups • Intervention follow-up interviews	• #/% with internet access • # reported technology issues	• Categorized technology needs • Participant reports of technology issues • CHW/HCN and health coach reports
Community members have access to healthy food and safe spaces for physical activity	• Platform engagement data • Pilot focus groups • Intervention follow-up interviews • Physical activity surveys • Food security surveys	• #/% physical activity logged • Δ food security indicators • Δ physical activity indicators	• Participant reports
Community members have access to their own health data	• Platform engagement data • Biometric assessments	• # weekly weigh-ins • Δ weight • Δ BMI • Δ HbA1C • Δ blood pressure	• Participant reports
CHW is able to identify relevant social needs	• Health-related social needs screening tool • Pilot focus groups • Intervention follow-up interviews	• # needs identified • Type of needs identified	• Categorized SDOH needs • Participant reports of SDOH needs
CHW is able to facilitate access to address social needs	• CHW / HCN referral data	• # referrals generated • #/% referrals successfully linked to services	• Categorized SDOH needs by referral status • Participant reports of SDOH needs

^1^Abbreviations: CHW - Community Health Worker; HCN - Hospital-based Community Nur**se**

#### Pilot results & adaptations

Three CCPs specifically informed programmatic changes for the full feasibility study. Pilot findings and corresponding program adaptations are summarized in Table 4.

**Table 4:** Summary of virtual Diabetes Prevention Program intervention pilot findings across data sources

Critical Quality Control Point	Finding	Supporting Data (survey results, platform engagement data, illustrative quotes, implementation tracker)	Program Adaptations
Community members engage and participate	Some participants found the program enrollment process confusing or overwhelming	*Implementation tracker*	- Welcome packet developed outlining steps and roles. - Program enrollment process unbundled to take place over several weeks.
	Participants felt that the range of program services promoted accountability	*Illustrative quotes* “So when all the good stuff that you all put together, it made me feel accountable. I was up there every day, ‘You got to do this. You can do this’” “having the contact like…the weekly contact [and] the text messages about exercises and meal plans and stuff from a center having that stuff right in your face, for me that absolutely helps” “I’ll agree with having that kind of voice there, to chime in every once in a while to be like, Hey how’s it going? Are you doing this? Are you doing that? …and if I don’t have that kick, I don’t do stuff. So it’s nice having that constant reminder to try to remind myself like, oh, I shouldn’t eat that or I shouldn’t drink this. But everything is easy. And it’s nice having that little kind of voice in the back of your head almost.”	- Continued primary program components
	Participants valued support and expertise from health coaches and CHWs/HCNs	*Illustrative quotes* “Each one of them was very generous with their time and information…they always made it clear that they’re available to me” “I have good experience with them. They’re very kind, very helpful, very encouraging, and I just love that even when sometimes I don’t feel encouraged, especially when my breathing acts up, and then I can’t do exercise, and it’s just crazy. But they were very encouraging to me”	Continued inclusion of health coach and CHW/HCN role
	Some participants had low engagement in platform webinars due to time constraints, even if they liked the content.	*Illustrative quotes* Facilitator 1: Do you remember doing anything like that? But it was within the Incenta Health app when you go to watch your webinar each week. R2: I don’t remember that at all? R5: I think I forgot do this. R4: Yeah, me either. R1: Me too. R3: This is the first I’m hearing of it. “I have watched [the webinars], I didn’t really get to watch a lot myself. But I did like them. I enjoyed them. “	Introductory webinar (Session 0) instituted. Strengthened CHW and health coach encouragement and support for participant to use platform and watch webinars together
	Some participants felt discouraged during weigh-ins, especially those that did weigh-ins more frequently than the program required (e.g., daily)	*Illustrative quotes* I didn’t like the scale weighing in the morning. I didn’t like [it] because one day it told me one thing, and the next day, it told me something else. I was upset. “I would get weighed in every morning…They did say I lost weight, I was happy. But when I [saw] the next day I gained it back, I wanted to throw that scale out the window.	Health coaches to add encouragement not to weigh in more than weekly.
	Some participants used the wearable activity tracker in place of the vDPP platform	*Illustrative quotes* “Part that I liked about it the most and the part that would be really interesting to delve further into is the [wearable activity tracker] app, because there’s a lot of videos that you can follow, there’s challenges that you could join and it’s just a big motivator, much more than I didn’t get worked into the [platform] app.” “So I set those goals on the Fitbit and it just made it a lot easier to be able to keep up with both, to keep track things on both apps was just too much.”	Delayed wearable activity tracker until completion of one-year program
	Participant felt confusion about CHW/HCN and health coach roles	*Implementation tracker* *Illustrative quotes* “I just think that it was a lot of contact with people. If it could just be one person that you report to, I know everyone has different roles in different areas, so I was just wishing. I was confused about everybody that I had to correspond with throughout the whole time. “I agree, the different people calling. It was like I said, one person all the time, because you talk to the one person and you tell them something, and then you do it again to another. One person will be good.”	Welcome packet developed with clarification of program roles, with names and phone numbers.
	Some participants struggled to do the body weight exercises suggested on the platform	*Illustrative quotes* “I didn’t do it. I couldn’t do the floors either. I mean, I walk, but I just couldn’t do those floor exercises.” “I’ve had a problem with the exercise because I’m dealing with some pretty bad back issues which kind of limits what I’m able to do. So that was a big thing for me not being able to do certain exercises.”	Health coaches will let study participants know that exercise modifications are available
	Some participants were interested in more peer support	*Illustrative quotes* “It’d be wonderful to have ladies who can get together and be a big support to one another that I know that we all can make it through this in losing weight.” So I feel like the other ladies walking together, live in the same area, and get to meet each other, and we can just be walking, bring our waters and walk.” “I like doing like in-person things, I’m a social butterfly. I like to be around people. So I mean, I would like [to] have that option to be like if you wanted to meet with somebody once a week or something, or maybe even when it comes to exercises, because I know not everybody can afford like a gym membership or something.”	CHWs/HCNs and health coaches will provide and encourage opportunities for peer connection through the platform chat feature.
Community members receive IT/website navigation support from health coach & CHW	Some participants had difficulty setting up platform smart scale^2^	*Implementation tracker* *Illustrative quotes* “So when I would weigh myself, it wasn’t keeping track of my weight. I had to do it manually. I ended up writing it down every day or putting it in the Fitbit.” “I got on the scale and I went to take the picture once a week and it wasn’t working. It just wasn’t syncing up with my phone. So I sent that one back. [health coach] sent me out a new one, same thing was happening.”	Introductory webinar (Session 0) instituted. Welcome packet developed with a section on setting up and using the platform smart scale
	Some participants had trouble setting up their vDPP platform accounts	*Implementation tracker*	Introductory webinar (Session 0) instituted. Welcome packet developed with a section on setting up vDPP platform accounts
	Participants found the platform easy to use after enrollment	*Participant satisfaction survey* 90% (*n* = 9) *agreed* or *strongly agreed* that the app was easy to use and understand 100% (*n* = 10) *agreed* or *strongly agreed* that tracking physical activity on the app was easy	Continued platform use
CHW is able to identify relevant social needs	Many participants had relevant social needs that could be addressed by a CHW	*Health-related social needs screening tool* stress (*n* = 8); food insecurity (*n* = 7); transportation (*n* = 5); housing (*n* = 4), utility bills (*n* = 4), social support (*n* = 4), behavioral health (*n* = 4), employment (*n* = 2); education (*n* = 1) *Illustrative quotes* “I had a nice one too with the resources like food banks information, and she gave me a lot of helpful information to help me” “I can’t forget her, because the situation that I’m going through right now… it’s just crazy and it’s stressful. And she did help me out with a lot of resources and finding stuff for me to call and stuff.”	Continued use of health-related social needs screening tool at baseline to identify relevant social needs.
	Some participants shared relevant social needs with other support staff, such as health coaches.	*Implementation tracker*	Weekly check-ins established between CHWs/HCNs and health coaches.

^1^ Abbreviations: CHW - Community Health Worker; HCN - Hospital-based Community Nurse

^2^The platform smart scale was provided by the platform to log weekly weigh-ins on the platform application

### CCP 1: Community members engage & participate

#### Feedback

We identified several program strengths that would likely facilitate engagement and participation. Pilot participants described how the program promoted a sense of accountability through daily text messages, weekly check-ins from health coaches and CHWs, weigh-ins, and the wearable fitness device. They appreciated the CHW and HCN for their support, expertise, and empathy. Participants noted that this role was especially important when they felt that they did not have support from family or friends for lifestyle change in their personal life.

We also identified some barriers to program engagement. Pilot participants had varying levels of platform engagement in the three required program components (webinars, weekly weigh-ins, and weekly physical activity logs). There was low engagement with the webinar content, with four of 11 participants (36.3%) completing no webinars and another four (36.3%) completing one out of five webinars. Two participants (18.2%) completed all five webinars, and one participant (9.1%) completed three webinars. Some participants described forgetting to watch the webinars, while others skipped them due to time constraints.

Engagement was higher with the weekly weigh-ins, with six participants (54.5%) completing four to five weigh-ins, three participants (27.3%) completing two to three weigh-ins, one participant (9.1%) completing one weigh-in, and one participant (9.1%) completing no weigh-ins. Participants shared mixed feelings about the weekly weigh-ins, with some participants appreciative of the element of accountability and others discouraged when they did not see their desired results. This was especially true for participants who initially weighed themselves more frequently than required (e.g., daily). Some participants also experienced challenges completing some of the home exercises suggested on the platform due to described physical limitations.

Though participants liked the wearable activity tracker, several participants described using it in place of the vDPP application, potentially limiting engagement in key program components. This was evidenced in the physical activity logs, where three participants completed four to five entries, four participants completed two to three entries, one participant completed one entry, and three participants completed no entries. Detailed platform engagement data is available in Additional File #10.

In addition to platform engagement barriers, participants also described some confusion about support staff roles, especially in distinguishing between the health coach and CHW/HCN. Some participants expressed frustration that they felt they were repeating themselves to different staff members.

#### Adaptations

We made several adaptations to help promote program facilitators and address barriers in program engagement. First, we modified components of the initial enrollment process to reduce role confusion and the sense of hurriedness. We developed a new participant welcome packet that included an enrollment timeline, a description of program roles, and instructions on navigating the platform application and smart scale. The CHW/HCNs would review the welcome packet with participants during their first meeting. Additionally, we extended the enrollment timeline from one week to four weeks and spaced out each task. This week-by-week timeline was outlined for participants in the welcome packet.

We also made some adaptations to the program delivery. To promote platform engagement, the CHW/HCNs and health coaches would offer to watch the first webinar with participants. This was intended to both facilitate initial engagement, given that approximately one third of pilot participants did not watch any webinars, and to promote engagement with the content that could facilitate watching future webinars. They also advised participants not to weigh in more than weekly and offered adaptations for home exercises. To address participants’ suggestions about peer support, the CHW will share contact information of local participants upon request and health coaches will encourage participants to use the group chat function on the platform. Lastly, in response to focus group findings that some participants used the wearable activity tracker application in place of the vDPP platform, we delayed the provision of the device to the end of the yearlong program.

### CCP 2: Community members receive IT/website support from CHW/HCN and health coach

#### Feedback

The main barrier pilot participants identified to using the platform was the initial enrollment and set-up process, identified through the implementation tracker and post-pilot focus groups. Some participants struggled to set up their smart scale provided by the platform and to complete initial enrollment on the platform. The CHWs/HCNs noted through the implementation tracker that the platform enrollment process differed between the mobile application and web browser, leading to some additional confusion. After enrollment was completed, pilot participants generally found the vDPP platform easy to use, with at least 90% of surveyed participants selecting *agreed* or *strongly agreed* with positively framed statements about the ease of the platform use, tracking physical activity, and the platform meal plan. Full participant satisfaction survey results are available in Additional File #11.

#### Adaptations

Based on these barriers with platform enrollment and scale set-up, we made two key adjustments to facilitate the use of the vDPP platform (see Table 4). First, we included in the welcome packet detailed step-by-step instructions for platform enrollment and setting up the smart scale. Next, we added a “Session Zero”, or introductory session, with platform health coaches to familiarize participants with platform enrollment and program navigation and provide an opportunity to ask questions. These sessions were planned to be recorded to be accessible to all participants. “Session 0” was also intended to address low engagement in the platform components (described under CCP 1) by facilitating familiarity with the platform.

### CCP 3: CHW is able to identify relevant social needs

#### Feedback

We identified several areas of social needs where a CHW/HCN could provide support. First, on the baseline health-related social needs screener, the CHW/HCN identified pilot participant needs related to stress (*n* = 8), food insecurity (*n* = 7), lack of transportation (*n* = 5), housing (*n* = 4), utility bills (*n* = 4), social support (*n* = 4), behavioral health (*n* = 4), employment (*n* = 2), and education (*n* = 1) (See Additional File #12 for complete health-related social needs screening results). On the participant satisfaction survey, 50% of pilot participants (*n* = 5) said that the cost of food kept them from participating in every part of the program sometimes or frequently. Additionally, 50% of participants (*n* = 5) cited health issues and 30% of participants cited family responsibilities as preventing full program participation (see *Additional File #11* for complete participant satisfaction survey results).

#### Adaptations

As described in CCP 1, a key barrier to identifying needs was unclear program roles leading to inconsistent participant communication with the CHW/HCN. In addition to the adaptations described for CCP 1, to help facilitate the CHW’s ability to identify relevant social needs, we established weekly check-ins between research staff, health coaches, and the CHW/HCN to discuss participant issues and troubleshoot program barriers and facilitators for individual participants. This enabled the health coach and research staff to share issues identified by participants that could be best addressed by the CHW/HCN.

## Discussion

We used a best-practices [31, 42] community-engaged approach to design a virtual Diabetes Prevention Program (vDPP) intervention in two communities with low incomes at higher risk for diabetes. Our initial design adapted the DPP through virtual delivery with asynchronous programming and individual health coaching as well as the addition of a care coordination component. Formative interviews showed that communities of focus accepted the vDPP concept and delivery mechanism and felt care coordination could be beneficial. Through the use of the PIP analysis, we identified key programmatic components and critical quality control points (CCPs) to measure in the pilot research phase. Finally, we conducted a pilot study to inform program adaptations and improve implementation quality. We were able to do so by addressing key CCPs identified with the program impact pathway process, including ways to improve participant engagement and participation, health coach and CHW technology support, and CHW support in identifying relevant social needs.

As far as we know, this is the first study to demonstrate a step-by-step community-engaged design process with a quality improvement analysis to adapt the evidence-based National Diabetes Prevention Program for use in communities with low incomes at increased risk for diabetes. Our study is also the first to add a community care coordination component to the core vDPP to help address participant health-related social needs that may inhibit program engagement. This component is especially important given the potential role of direct and indirect significant life stressors and barriers such as cost of food, housing, transportation, caretaking responsibilities, and employment contributing to DPP attrition in similar communities [26, 58]. Our findings support the results of earlier vDPP feasibility studies, conducted with a different platform in similar communities, regarding program acceptability, interest, and the importance of both direct program support and peer support [59]. The full feasibility study evaluates the implementation of this vDPP intervention using a similar mixed-methods, iterative process as well as biometric and health outcomes. The full study was also designed to monitor the impact of adaptations made during the pilot, such as those to address the relatively low platform engagement and confusion in support staff roles, and implement and track any further program delivery changes made.

In addition to the implementation findings reported here, the pilot focus group analysis yielded preliminary contextual themes related to societal and personal beliefs on the relationship between diet, behavior change, and weight loss. Many participants described their program experience in the context of personal willpower and willingness to make dietary and lifestyle changes, often using self-stigmatizing terminology to describe perceived failures. More research is needed to explore the role of these beliefs in vDPP program engagement and participation, especially given the potential relationship between reduced self-stigma and weight loss and the lack of evidence for personal agency as the main factor determining for body weight [60–62]. 

This study had several limitations. First, this research was conducted during 14 months of the COVID-19 pandemic, resulting in remote data collection and corresponding recruitment challenges. For this reason, we could only include participants who could access the needed technology, reducing the applicability of our results to participants with less technology access or comfort. The platform selection team underwent a rigorous search among many virtual platforms available at that time. While they chose one that most closely addressed many of the participation barriers community members described in other lifestyle change programs, it was not fully representative of our communities of focus. The selected platform was only available in English, restricting participation of Spanish-speaking community members who represent a sizable demographic in the study communities and limiting the applicability of our results. Additionally, there was low cultural and ethnic representation among platform health coaches and webinar speakers available on the platform, which may have contributed to relatively low participant engagement and feelings of receiving personalized or relevant information. Future versions of the vDPP should be developed with broader demographic and linguistic representation. Lastly, the study’s geographical focus may limit the generalizability of our findings to other regions or areas with differing socio-economic and health profiles.

## Conclusion

By pairing the PIP quality improvement framework with a community-engaged approach to formative implementation science research, we developed a novel health promotion intervention for use in the planned implementation science study on the feasibility of a vDPP among communities with low incomes and at high risk for diabetes. This iterative process to intervention development will continue to be applied in the ongoing full feasibility study. Our innovative quality improvement methodology will be widely disseminated for broader learnings and applications.

## Supplementary Information

Below is the link to the electronic supplementary material.

Supplementary Material 1

Supplementary Material 2

Supplementary Material 3

Supplementary Material 4

Supplementary Material 5

Supplementary Material 6

Supplementary Material 7

Supplementary Material 8

Supplementary Material 9

Supplementary Material 10

Supplementary Material 11

Supplementary Material 12

## Data Availability

No datasets were generated or analysed during the current study.

## Abbreviations

CCP: Critical Quality Control Point
DPP: Diabetes Prevention Program
PIP: Program Impact Pathway
vDPP: Virtual Diabetes Prevention Program
Y-G PRC: Yale Griffin Prevention Research Center
