# Supplementary material for: Community-engaged design of a virtual diabetes prevention program intervention with care coordination
Source: BMC Health Serv Res. 2026 Jun 13;26:1166. doi: 10.1186/s12913-026-14860-0 (PMC13491910; doi:10.1186/s12913-026-14860-0)
Supplement: Supplementary file 1 [file 12913_2026_14860_MOESM10_ESM.docx]

Additional File

Virtual Diabetes Prevention Program Platform Engagement Data for Pilot Participants, April-May 2021

Figure S2

Webinar Engagement Over 5 Weeks

| Number of Webinars Watched | Number of Participants |
| --- | --- |
| 0 | 4 |
| 1 | 4 |
| 2 | 0 |
| 3 | 1 |
| 4 | 0 |
| 5 | 2 |

Figure S3

Weigh-in Engagement Over 5 Weeks

| Number of Weekly Weigh-ins | Number of Particpants |
| --- | --- |
| 0 | 1 |
| 1 | 1 |
| 2 | 1 |
| 3 | 2 |
| 4 | 5 |
| 5 | 1 |

Figure S4

Physical Activity Engagement Over 5 Weeks

| Number of Weeks with Physical Activity Data | Number of Participants |
| --- | --- |
| 0 | 3 |
| 1 | 1 |
| 2 | 3 |
| 3 | 1 |
| 4 | 1 |
| 5 | 2 |
