# Supplementary material for: Community-engaged design of a virtual diabetes prevention program intervention with care coordination
Source: BMC Health Serv Res. 2026 Jun 13;26:1166. doi: 10.1186/s12913-026-14860-0 (PMC13491910; doi:10.1186/s12913-026-14860-0)
Supplement: Supplementary file 2 [file 12913_2026_14860_MOESM11_ESM.docx]

Additional File #11

Participant Satisfaction Survey Results for Participants of 5-week vDPP Pilot

| **Table S3. Program Experience (N=10)** | | | | | | | |
| --- | --- | --- | --- | --- | --- | --- | --- |
|  | Strongly Disagree | Disagree | Agree | Strongly Agree | **Percent “Agree” or** **“Strongly Agree”** | **Mean (SD)** | **Range** |
|  | 1 | 2 | 3 | 4 |  |  |  |
| Overall, the app was easy to use and understand. (n=10) | 0 | 1 | 6 | 3 | 90.0% | 3.20 (0.63) | [2-4] |
| Tracking physical activity on the app was easy. (n=9) | 0 | 0 | 6 | 3 | 100% | 3.33 (0.50) | [3-4] |
| The meal plan on the app was easy to follow. (n=10) | 0 | 1 | 5 | 4 | 90.0% | 3.30 (0.67) | [2-4] |
| The health coach helped me to meet my goals. (n=10) | 0 | 1 | 6 | 3 | 90.0% | 3.20 (0.63) | [2-4] |
| The program motivated me to exercise more. (n=10) | 0 | 1 | 4 | 5 | 90.0% | 3.40 (0.70) | [2-4] |
| The program motivated me to change my diet. (n=9) | 0 | 1 | 5 | 3 | 88.9% | 3.22 (0.67) | [2-4] |
| I was able to get help for challenges on healthy eating and meal planning. (n=10) | 0 | 0 | 7 | 3 | 100% | 3.30 (0.48) | [3-4] |
| I learned more about healthy eating through this program. (n=10) | 0 | 0 | 4 | 6 | 100% | 3.60 (0.52) | [3-4] |
| I was able to get help for challenges on physical activity. (n=10) | 0 | 1 | 6 | 3 | 90.0% | 3.20 (0.63) | [2-4] |
| I learned more about the benefits of physical activity through this program. (n=10) | 0 | 0 | 6 | 4 | 100% | 3.40 (0.52) | [3-4] |
| I would choose an online diabetes prevention program again, over an in-person program. (n=10) | 0 | 0 | 7 | 3 | 100% | 3.30 (0.48) | [3-4] |
| I learned more about how to prevent diabetes through this program. (n=10) | 0 | 0 | 7 | 3 | 100% | 3.30 (0.48) | [3-4] |
| I would recommend this program to a family member or friend. (n=10) | 0 | 0 | 6 | 4 | 100% | 3.40 (0.52) | [3-4] |
| This program helped lower my risk of diabetes. (n=10) | 0 | 0 | 6 | 4 | 100% | 3.40 (0.52) | [3-4] |

| **Table S4. Barriers to Program Engagement (N=10)** | | | | |
| --- | --- | --- | --- | --- |
|  | **Yes, n (%)** | **Rarely, n (% of yes)** | **Sometimes, n (% of yes)** | **Frequently, n (% of yes)** |
| Has the **weather** kept you from completing some parts of the program? (n=10) | 3 (30.0%) | 0 | 3 (100%) | 0 |
| Has a **lack of transportation** kept you from completing some parts of the program? (n=10) | 0 (0%) | - | - | - |
| Have **health issues** kept you from completing some parts of the program? (n=10) | 5 (50.0%) | 0 | 3 (60.0%) | 2 (40.0%) |
| Has your **work** kept you from completing some parts of the program? (n=10) | 0 (0%) | - | - | - |
| Have **family activities**, like caring for children or other family members, kept you from completing some parts of the program? (n=10) | 3 (30.0%) | 0 | 2 (66.7%) | 1 (33.3%) |
| Has the **cost of food** kept you from completing some parts of the program? (n=10) | 5 (50.0%) | 0 | 3 (60.0%) | 2 (40.0%) |
| Have **community activities** keep you from completing some parts of the program? (n=10) | 0 (0%) | - | - | - |

| **Table S5. Time Commitment Involved with Program Components, N=10** | | | |
| --- | --- | --- | --- |
|  | **Too much, n (%)** | **Just right, n (%)** | **Not enough, n (%)** |
| Amount of time spent planning meals. (n=10) | 2 (20.0%) | 7 (70.0%) | 1 (10.0%) |
| Amount of time spent preparing and cooking meals. (n=10) | 1 (10.0%) | 8 (80.0%) | 1 (10.0%) |
| Amount of time spent tracking food on the app. (n=10) | 4 (40.0%) | 5 (50.0%) | 1 (10.0%) |
| Amount of time spent engaging in physical activity. (n=10) | 0 (0%) | 6 (60.0%) | 4 (40.0%) |
| Amount of time spent tracking physical activity on the app. (n=9) | 0 (0%) | 8 (88.9%) | 1 (11.1%) |
| Amount of time watching incentaHEALTH videos. (n=10) | 2 (20.0%) | 6 (60.0%) | 2 (20.0%) |
| Amount of time spent consulting with online coaches. (n=10) | 1 (10.0%) | 8 (80.0%) | 1 (10.0%) |
| Amount of time spent consulting with health professionals (community health workers/community health nurses, etc.) (n=10) | 0 (0%) | 9 (90.0%) | 1 (10.0%) |
