# Supplementary material for: Community-engaged design of a virtual diabetes prevention program intervention with care coordination
Source: BMC Health Serv Res. 2026 Jun 13;26:1166. doi: 10.1186/s12913-026-14860-0 (PMC13491910; doi:10.1186/s12913-026-14860-0)
Supplement: Supplementary file 3 [file 12913_2026_14860_MOESM12_ESM.docx]

Additional File #12

Health-Related Social Needs Screening Results for vDPP Intervention Pilot Participants

| **Table S6. Needs Identified by SDOH Screening, New Haven (N=5)** | |
| --- | --- |
|  | Identified on Tool, n (%) |
| Housing Need | 1 (20.0%) |
| Unlivable Conditions | 1 (20.0%) |
| Food Security | 3 (60.0%) |
| Lack of Transportation | 3 (60.0%) |
| Difficulties with Utilities | 0 (0%) |
| Employment Need | 0 (0%) |
| Education Need | 1 (20.0%) |
| Social Support Need | 3 (60.0%) |
| Stress Support Need | 3 (60.0%) |
| Behavioral Health Services Need | 2 (40.0%) |
| COVID-19 Need | 3 (60.0%) |
| COVID-19 Testing Information | 1 (20.0%) |
| COVID-19 Vaccine Information | 1 (20.0%) |
| Digital Connectivity Need | 0 (0%) |

| **Table S7. Needs Identified by SDOH Screening, The Valley (N=6)** | |
| --- | --- |
|  | Frequency (%) |
| Housing Need | 3 (50.0%) |
| Unlivable Conditions | 3 (50.0%) |
| Food Security | 4 (66.7%) |
| Lack of Transportation | 2 (33.3%) |
| Difficulties with Utilities | 4 (66.7%) |
| Employment Need | 2 (33.3%) |
| Education Need | 0 (0%) |
| Social Support Need | 1 (16.7%) |
| Stress Support Need | 5 (83.3%) |
| Behavioral Health Services Need | 2 (33.3%) |
| COVID Need | 3 (50.0%) |
| COVID Testing Information | 0 (0%) |
| COVID Vaccine Information | 0 (0%) |
| Digital Connectivity Need | 0 (0%) |
