# Supplementary material for: Community-engaged design of a virtual diabetes prevention program intervention with care coordination
Source: BMC Health Serv Res. 2026 Jun 13;26:1166. doi: 10.1186/s12913-026-14860-0 (PMC13491910; doi:10.1186/s12913-026-14860-0)
Supplement: Supplementary file 6 [file 12913_2026_14860_MOESM3_ESM.docx]

Additional File #3

Qualitative Interview and Focus Group Discussion Guides

Provider Interview Guide

1. Have you [or your organization] ever been involved in offering a Diabetes Prevention Program or other programs that promote healthy eating or physical activity to adults with limited resources? [IF SO] Please tell me about your experiences with offering [this program/these programs].

2. Have you ever referred adults with limited resources to a Diabetes Prevention Program or other programs that promote healthy eating or physical activity?

[IF SO] Please tell me about your experiences in referring them.

3. What are your first impressions of this program?

4. Would you refer someone to a virtual diabetes prevention program? Why or why not?

5. What challenges do you think we might face in conducting this program with residents?

6. What do you think is the best way to reach residents to recruit them into our study?

7. In this virtual diabetes prevention program, adults are asked to participate online at least once a week for four months. After that, they have to check in once a month for eight months. How would you recommend keeping people engaged in the program?

8. Is there anything you’d like to share about diabetes prevention in the community that we haven’t talked about yet?

9. Is there anyone else who you suggest we also interview as part of this study?

Resident Interview Guide

1. Have you [or your organization] ever been involved in offering a Diabetes Prevention Program or other programs that promote healthy eating or physical activity to adults with limited resources? [IF SO] Please tell me about your experiences with offering [this program/these programs].

2. Have you ever referred adults with limited resources to a Diabetes Prevention Program or other programs that promote healthy eating or physical activity?

[IF SO] Please tell me about your experiences in referring them.

3. What are your first impressions of this program?

4. Would you refer someone to a virtual diabetes prevention program? Why or why not?

5. What challenges do you think we might face in conducting this program with residents?

6. What do you think is the best way to reach residents to recruit them into our study?

7. In this virtual diabetes prevention program, adults are asked to participate online at least once a week for four months. After that, they have to check in once a month for eight months. How would you recommend keeping people engaged in the program?

8. Is there anything you’d like to share about diabetes prevention in the community that we haven’t talked about yet?

9. Is there anyone else who you suggest we also interview as part of this study?

Focus Group Discussion Guide

The Yale-Griffin Prevention Research Center pilot study has included an online program, incentaHEALTH Healthy Me, access to a health coach, and work with a community health worker (New Haven) / community health nurse (Lower Naugatuck Valley). What have you thought of the program based on your experience during the past four weeks?

What have you liked best about the pilot program? Why?

What have you liked least about the pilot program? Why?

What would you change about the pilot program?

Diabetes prevention programs emphasize personal support. What has been your experience with the personal support in the pilot program?

Have you changed any of your health habits during the pilot program? If so, how?

Our study includes people from many different backgrounds, races, ethnicities, and cultures, and experiences. How, if at all, could the incentaHEALTH Healthy Me program be made more inclusive?

We know that being healthy can mean different things. Do you feel like the program helped you to be healthy? How so?

Is there anything you would like to add that we haven’t talked about yet?
