# Supplementary material for: Community-engaged design of a virtual diabetes prevention program intervention with care coordination
Source: BMC Health Serv Res. 2026 Jun 13;26:1166. doi: 10.1186/s12913-026-14860-0 (PMC13491910; doi:10.1186/s12913-026-14860-0)
Supplement: Supplementary file 7 [file 12913_2026_14860_MOESM4_ESM.docx]

Additional File 4

In-depth Interview Participant Characteristics

| Community Residents Interview Participant Characteristics | | |
| --- | --- | --- |
| *Site* | ***n*** | ***%*** |
| New Haven | 10 | 48% |
| Ansonia or Derby | 11 | 52% |
| Total | **21** | **100%** |
| *Diabetes Health Status* | ***n*** | ***%*** |
| Met CDC criteria for prediabetes | 19 | 90% |
| Diagnosed with Type 1 diabetes | 0 | 0% |
| Diagnosed with Type 2 diabetes | 2 | 10% |
| Total | **21** | **100%** |

| Community Provider Interview Participant Characteristics | | |
| --- | --- | --- |
| *Site* | ***n*** | ***%*** |
| New Haven | 5 | 38% |
| Ansonia or Derby | 8 | 62% |
| Total | **13** | **100%** |
| *Affiliation* | ***n*** | ***%*** |
| Housing authority | 5 | 38% |
| Community health worker or community health nurse | 3 | 23% |
| Program manager or diabetes educator | 2 | 15% |
| Physician | 1 | 8% |
| Community leader | 1 | 8% |
| Health district | 1 | 8% |
| Total | **13** | **100%** |
