# Supplementary material for: Community-engaged design of a virtual diabetes prevention program intervention with care coordination
Source: BMC Health Serv Res. 2026 Jun 13;26:1166. doi: 10.1186/s12913-026-14860-0 (PMC13491910; doi:10.1186/s12913-026-14860-0)
Supplement: Supplementary file 8 [file 12913_2026_14860_MOESM5_ESM.docx]

Additional File 5

Participant Technology Survey

**Participant ID:_______________**

**City: ______________________**

**Date: ______________________**

1. **Do you have internet access in your home?**

€ Yes

€ No

€ Unsure

2. **a.[IF 1 = YES] What type of internet access do you have at home? Check all that apply.**

€ I use my Wi-Fi network (like Xfinity or Frontier)

€ I use the data plan from my phone (with limited data)

€ I use the unlimited data plan from my phone

€ Other (please describe): ____________________________

€ Not sure

€ I do not have internet access in my home.

**b. [IF 1 = NO or 1 = UNSURE] Do you access the internet outside of your home?**

€ Yes

€ No [SKIP TO QUESTION 4]

€ Unsure

**c. [IF 2b = YES] Where do you usually access the internet outside of your home? Select all that apply.**

€ At work

€ At a friend or family member’s house

€ At the library or other public place

€ Other, please describe: ________________

3. **How often do you usually access the internet? You can count times you use a phone, a tablet, a computer, or other device.**

€ Several times a day

€ Once or twice a day

€ A few times a week

€ Once a week

€ Less than once a week

€ Never

4. **How comfortable are you accessing the internet on your own?**

€ Very comfortable

€ Somewhat comfortable

€ Not very comfortable

€ I do not access the internet on my own

5. **Do you live with someone who can help you access the internet?**

€ Yes

€ No

6. **How often do you use each of the following devices to access the internet?**

**Check one response for each line.**

|  | Never | Sometimes | Often | Always |
| --- | --- | --- | --- | --- |
| A computer that I own | € | € | € | € |
| A computer in a shared or public space (library, community room, etc.) | € | € | € | € |
| A computer at a friend’s or family member’s home | € | € | € | € |
| A smartphone or tablet that I own | € | € | € | € |
| A smartphone or tablet owned by a friend or family member | € | € | € | € |
| Other type of mobile phone (please specify): | € | € | € | € |
| Other (please describe): | € | € | € | € |

7. **Have you ever used a computer or smartphone to track your diet or exercise?** For example, websites, apps, or wearable devices like Fitday.com, MyFitnessPal, Fooducate, RunKeeper, FitBit, Noom, Apple Watch, etc.

€ Yes, but I don’t use them now

€ Yes, I currently use one or more of these

€ No

8. **What is your access to the internet like?** Does anything make it difficult for you to access the internet? (For example, is it reliable? Do you have enough data?)

   _______________________________________________________________________

   _______________________________________________________________________

***Thank you for completing this survey!***
