# Supplementary material for: Community-engaged design of a virtual diabetes prevention program intervention with care coordination
Source: BMC Health Serv Res. 2026 Jun 13;26:1166. doi: 10.1186/s12913-026-14860-0 (PMC13491910; doi:10.1186/s12913-026-14860-0)
Supplement: Supplementary file 10 [file 12913_2026_14860_MOESM7_ESM.docx]

Additional File #7

vDPP Pilot Intervention Health-Related Social Needs Screening Tool

ID Participant Identifiers

- Study ID # (1) __________________________________________________
- Full Name (2) __________________________________________________
- Town of Residence (3) __________________________________________________
- Name of CHW/HCN (4) __________________________________________________

Introductory Script Introduction to the screening tool   *As part of our conversation today, we'd like to ask you a few questions about accessing community and social resources.* 
 *By answering these questions, we may be able to connect you to services or programs that are available for you and your family, including housing, food and transportation.*
 *Some of the questions are personal, but please know we will keep your answers confidential and use them to provide you with any resources we can.*
 *If at any time you feel uncomfortable, you can also opt out of a question. Does that sound okay?*

End of Block: Introductory Script

Start of Block: Living Situation

H1 **Living Situation**

|  |  |
| --- | --- |

Q1 What is your living situation today?

- I have a steady place to live (1)
- I have a place to live today, but I am worried about losing it in the future (2)
- I do not have a steady place to live (I am temporarily staying with others, in a hotel, in a shelter, living outside on the street, on a beach, in a car, abandoned building, bus or train station, or in a park) (3)

|  |  |
| --- | --- |

Q2 Think about the place you live. Do you have problems with any of the following? [CHOOSE ALL THAT APPLY]

- Pests such as bugs, ants, or mice (1)
- Mold (2)
- Lead paint or pipes (3)
- Lack of heat (4)
- Oven or stove not working (5)
- Smoke detectors missing or not working (6)
- Water leaks (7)
- ⊗None of the above (8)

End of Block: Living Situation

Start of Block: Food

H2 **Food**

Script *The following questions related to food may be similar to questions you have already answered during the study. We want to thank you for taking the time to answer these again.*   Some people have made the following statements about their food situation. Please answer whether the statements were OFTEN, SOMETIMES, or NEVER true for you and your household in the last 12 months.

Q3 Within the past 12 months, you worried that your food would run out before you got money to buy more.

- Often true (1)
- Sometimes true (2)
- Never true (3)

Q4 Within the past 12 months, the food you bought just didn't last and you didn't have money to get more.

- Often true (1)
- Sometimes true (2)
- Never true (3)

End of Block: Food

Start of Block: Transportation

H3 **Transportation**

Q5 In the past 12 months, has lack of reliable transportation kept you from medical appointments, meetings, work or from getting things needed for daily living?

- Yes (1)
- No (2)

End of Block: Transportation

Start of Block: Utilities

H4 **Utilities**

Q6 In the past 12 months, has the electric, gas, oil, or water company threatened to shut off services in your home?

- Yes (1)
- No (2)
- Already shut off (3)

End of Block: Utilities

Start of Block: Employment

H5 **Employment**

Q7 Do you want help finding or keeping work or a job?

- Yes, help finding work (1)
- Yes, help keeping work (2)
- I do not need or want help (3)

End of Block: Employment

Start of Block: Education

H6 **Education**

Q8 Do you want help with school or training? For example, starting or completing job training or getting a high school diploma, GED or equivalent.

- Yes (1)
- No (2)

End of Block: Education

Start of Block: Family and Community Support

H7 **Family and Community Support**

Q9 How often do you feel lonely or isolated from those around you?

- Never (1)
- Rarely (2)
- Sometimes (3)
- Often (4)
- Always (5)

Q10 Is there anyone who helps you with coordinating medical care or other services? [CHOOSE ALL THAT APPLY]

- Family member (1)
- Friend (2)
- Social Worker (3)
- Care Coordinator (4)
- Patient Navigator (5)
- Community Health Worker (6)
- Other (specify): (7) __________________________________________________
- ⊗None of the above (8)

End of Block: Family and Community Support

Start of Block: Mental Health

H8 **Mental Health**

Q11 Stress means a situation in which a person feels tense, restless, nervous, or anxious, or is unable to sleep at night because their mind is troubled all the time. Do you feel this kind of stress these days?

- Not at all (1)
- A little bit (2)
- Somewhat (3)
- Quite a bit (4)
- Very much (5)

Q12 Would you be interested in resources to help with any of the following? [CHOOSE ALL THAT APPLY]

- Mental/emotional health (e.g., counseling therapy) (1)
- Tobacco use / smoking cessation (2)
- Alcohol abuse (3)
- Drug abuse (4)
- ⊗Not interested in any of these services (5)

End of Block: Mental Health

Start of Block: COVID-19 Related Needs

H9 **COVID-19 Related Needs**

Q13 Overall, how much has the COVID-19 outbreak, and the resulting changes to daily life, affected your life?

- Not at all (1)
- A little (2)
- Somewhat (3)
- A lot (4)
- A great deal (5)

Skip To: Q15 If Overall, how much has the COVID-19 outbreak, and the resulting changes to daily life, affected yo... = Not at all

Skip To: Q15 If Overall, how much has the COVID-19 outbreak, and the resulting changes to daily life, affected yo... = A little

Skip To: Q15 If Overall, how much has the COVID-19 outbreak, and the resulting changes to daily life, affected yo... = Somewhat

Skip To: Q14 If Overall, how much has the COVID-19 outbreak, and the resulting changes to daily life, affected yo... = A lot

Skip To: Q14 If Overall, how much has the COVID-19 outbreak, and the resulting changes to daily life, affected yo... = A great deal

Q14 In what ways has COVID-19 affected you?

________________________________________________________________

Q15 Have you been able to effectively practice social distancing? Social distancing is defined as reducing your physical contact with people outside of your home in social, work, or school settings by avoiding large groups and staying 6 feet away from other people when out in public.

- Yes (1)
- No (2)

Q16 Do you know where you or your family members can get tested for COVID-19 in your community?

- Yes (1)
- No (2)

Q17 Have you received the COVID-19 vaccine?

- Yes (1)
- No (2)

Skip To: End of Block If Have you received the COVID-19 vaccine? = Yes

Q18 When a vaccine becomes available for you, would you be willing to get it?

- Yes (1)
- No (2)
- Unsure (3)

Skip To: End of Block If When a vaccine becomes available for you, would you be willing to get it? = No

Q19 If you are currently eligible to receive the COVID-19 vaccine, would you like assistance in scheduling your vaccine appointment?

- Yes (1)
- No (2)

End of Block: COVID-19 Related Needs

Start of Block: Communications & Digital Connectivity

H10 **Communications & Digital Connectivity**

Q20 Do you have access to technology for communicating with others and getting information electronically - in particular, a working computer/tablet/smartphone AND internet access/WiFi?

- Always (1)
- Sometimes (2)
- Never (3)

Q21 How would you describe your overall level of comfort using technology to communicate with others and get information electronically?

- Excellent (1)
- Very good (2)
- Good (3)
- Fair (4)
- Poor (5)

End of Block: Communications & Digital Connectivity

Start of Block: Screenings

Display This Question:

If What is your living situation today? = I have a place to live today, but I am worried about losing it in the future

Or What is your living situation today? = I do not have a steady place to live (I am temporarily staying with others, in a hotel, in a shelter, living outside on the street, on a beach, in a car, abandoned building, bus or train station, or in a park)

|  |  |  |
| --- | --- | --- |

Q1_1 Housing need

- Housing need (1)

Display This Question:

If Think about the place you live. Do you have problems with any of the following? [CHOOSE ALL THAT... = Pests such as bugs, ants, or mice

Or Think about the place you live. Do you have problems with any of the following? [CHOOSE ALL THAT... = Mold

Or Think about the place you live. Do you have problems with any of the following? [CHOOSE ALL THAT... = Lead paint or pipes

Or Think about the place you live. Do you have problems with any of the following? [CHOOSE ALL THAT... = Lack of heat

Or Think about the place you live. Do you have problems with any of the following? [CHOOSE ALL THAT... = Oven or stove not working

Or Think about the place you live. Do you have problems with any of the following? [CHOOSE ALL THAT... = Smoke detectors missing or not working

Or Think about the place you live. Do you have problems with any of the following? [CHOOSE ALL THAT... = Water leaks

|  |  |  |
| --- | --- | --- |

Q2_1 Unlivable conditions

- Unlivable conditions (1)

Display This Question:

If Within the past 12 months, you worried that your food would run out before you got money to buy m... = Often true

Or Within the past 12 months, you worried that your food would run out before you got money to buy m... = Sometimes true

Or Within the past 12 months, the food you bought just didn't last and you didn't have money to get... = Often true

Or Within the past 12 months, the food you bought just didn't last and you didn't have money to get... = Sometimes true

|  |  |  |
| --- | --- | --- |

Q3/4_1 Food insecurity

- Food insecurity (1)

Display This Question:

If In the past 12 months, has lack of reliable transportation kept you from medical appointments, me... = Yes

|  |  |  |
| --- | --- | --- |

Q5_1 Lack of transportation

- Lack of transportation (1)

Display This Question:

If In the past 12 months, has the electric, gas, oil, or water company threatened to shut off servic... = Yes

Or In the past 12 months, has the electric, gas, oil, or water company threatened to shut off servic... = Already shut off

|  |  |  |
| --- | --- | --- |

Q6_1 Difficulties with utilities

- Difficulties with utilities (1)

Display This Question:

If Do you want help finding or keeping work or a job? = Yes, help finding work

Or Do you want help finding or keeping work or a job? = Yes, help keeping work

|  |  |  |
| --- | --- | --- |

Q7_1 Employment need

- Employment need (1)

Display This Question:

If Do you want help with school or training? For example, starting or completing job training or get... = Yes

|  |  |  |
| --- | --- | --- |

Q8_1 Education need

- Education need (1)

Display This Question:

If How often do you feel lonely or isolated from those around you? = Often

Or How often do you feel lonely or isolated from those around you? = Always

|  |  |  |
| --- | --- | --- |

Q9_1 Social support need

- Social support need (1)

Display This Question:

If Stress means a situation in which a person feels tense, restless, nervous, or anxious, or is unab... = Quite a bit

Or Stress means a situation in which a person feels tense, restless, nervous, or anxious, or is unab... = Very much

|  |  |  |
| --- | --- | --- |

Q11_1 Stress support need

- Stress support need (1)

Display This Question:

If Would you be interested in resources to help with any of the following? [CHOOSE ALL THAT APPLY] = Mental/emotional health (e.g., counseling therapy)

Or Would you be interested in resources to help with any of the following? [CHOOSE ALL THAT APPLY] = Tobacco use / smoking cessation

Or Would you be interested in resources to help with any of the following? [CHOOSE ALL THAT APPLY] = Alcohol abuse

Or Would you be interested in resources to help with any of the following? [CHOOSE ALL THAT APPLY] = Drug abuse

|  |  |  |
| --- | --- | --- |

Q12_1 Behavioral health services need

- Behavioral health services need (1)

Display This Question:

If Overall, how much has the COVID-19 outbreak, and the resulting changes to daily life, affected yo... = A lot

Or Overall, how much has the COVID-19 outbreak, and the resulting changes to daily life, affected yo... = A great deal

|  |  |  |
| --- | --- | --- |

Q13_1 COVID need

- COVID need (1)

Display This Question:

If Do you know where you or your family members can get tested for COVID-19 in your community? = No

|  |  |  |
| --- | --- | --- |

Q16_1 COVID testing info need

- COVID testing info need (1)

Display This Question:

If If you are currently eligible to receive the COVID-19 vaccine, would you like assistance in sched... = Yes

|  |  |  |
| --- | --- | --- |

Q19_1 COVID vaccine info need

- COVID vaccine info need (1)

Display This Question:

If Do you have access to technology for communicating with others and getting information electronic... = Never

|  |  |  |
| --- | --- | --- |

Q20_1 Digital connectivity need

- Digital connectivity need (1)

End of Block: Screenings

Start of Block: Wrap-up Script

Wrap-up Script **Wrap-up Script** Thank you so much for answering these questions.

 ***[If participant screened positive to any of the questions above]*** It sounds like you could benefit from getting information or resources on:
   ${Q1_1/ChoiceGroup/SelectedChoices} ${Q2_1/ChoiceGroup/SelectedChoices} -${Q2/ChoiceGroup/SelectedChoices}
 ${Q3/4_1/ChoiceGroup/SelectedChoices} ${Q5_1/ChoiceGroup/SelectedChoices} ${Q6_1/ChoiceGroup/SelectedChoices} ${Q7_1/ChoiceGroup/SelectedChoices} ${Q8_1/ChoiceGroup/SelectedChoices} ${Q9_1/ChoiceGroup/SelectedChoices} ${Q11_1/ChoiceGroup/SelectedChoices} ${Q12_1/ChoiceGroup/SelectedChoices}    -${Q12/ChoiceGroup/SelectedChoices} ${Q13_1/ChoiceGroup/SelectedChoices}      -${Q14/ChoiceTextEntryValue} ${Q16_1/ChoiceGroup/SelectedChoices} ${Q19_1/ChoiceGroup/SelectedChoices} ${Q20_1/ChoiceGroup/SelectedChoices}   Is that correct?   Could we come up with a plan together to address this/these?   Are there any other pressing issues or needs that you may have at the moment that we have not discussed?   Do you have any questions for me?     CLICK ARROW BELOW TO SUBMIT RESPONSES.

End of Block: Wrap-up Script
