# Supplementary material for: Community-engaged design of a virtual diabetes prevention program intervention with care coordination
Source: BMC Health Serv Res. 2026 Jun 13;26:1166. doi: 10.1186/s12913-026-14860-0 (PMC13491910; doi:10.1186/s12913-026-14860-0)
Supplement: Supplementary file 11 [file 12913_2026_14860_MOESM8_ESM.docx]

Additional File #8

Participant Satisfaction Survey for vDPP Intervention Pilot Participants

H8_PW
We are interested in finding out about the experience you have had with the program and your satisfaction.

PS1_PW  Please rate how much you **agree or disagree** with the following statements:

|  | Strongly disagree (1) | Disagree (2) | Agree (3) | Strongly agree (4) |
| --- | --- | --- | --- | --- |
| Overall, the app was easy to use and understand. (PS1a_PW) |  |  |  |  |
| Tracking physical activity on the app was easy. (PS1b_PW) |  |  |  |  |
| The meal plan on the app was easy to follow. (PS1c_PW) |  |  |  |  |
| The health coach helped me to meet my goals. (PS1d_PW) |  |  |  |  |
| The program motivated me to exercise more. (PS1e_PW) |  |  |  |  |
| The program motivated me to change my diet. (PS1f_PW) |  |  |  |  |
| I was able to get help for challenges on healthy eating and meal planning (PS1g_PW) |  |  |  |  |
| I learned more about healthy eating through this program. (PS1h_PW) |  |  |  |  |
| I was able to get help for challenges on physical activity (PS1i_PW) |  |  |  |  |
| I learned more about the benefits of physical activity through this program (PS1j_PW) |  |  |  |  |
| I would choose an online diabetes prevention program again, over an in-person program. (PS1k_PW) |  |  |  |  |
| I learned more about how to prevent diabetes through this program. (PS1l_PW) |  |  |  |  |
| I would recommend this program to a family member or friend. (PS1m_PW) |  |  |  |  |
| This program helped lower my risk of diabetes. (PS1n_PW) |  |  |  |  |

| Page Break |  |
| --- | --- |

PS2I_PW The following questions ask about what may have kept you from completing parts of the program.

PS2_PW Has the **weather** kept you from completing some parts of the program?

- Yes (1)
- No (0)

Display This Question:

If Has the weather kept you from completing some parts of the program? = Yes

PS2a_PW How often did the **weather** keep you from completing some parts of the program?

- Never (1)
- Rarely (2)
- Sometimes (3)
- Often (4)

PS3_PW Has a **lack of transportation** kept you from completing some parts of the program?

- Yes (1)
- No (0)

Display This Question:

If Has a lack of transportation kept you from completing some parts of the program? = Yes

PS3a_PW How often did **lack of transportation** keep you from completing parts of the program?

- Never (1)
- Rarely (2)
- Sometimes (3)
- Often (4)

PS4_PW Have **health issues** kept you from completing some parts of the program?

- Yes (1)
- No (0)

Display This Question:

If Have health issues kept you from completing some parts of the program? = Yes

PS4a_PW How often did your **health issues** keep you from completing some parts of the program?

- Never (1)
- Rarely (2)
- Sometimes (3)
- Often (4)

PS5_PW Has your **work** kept you from completing some parts of the program?

- Yes (1)
- No (0)

Display This Question:

If Has your work kept you from completing some parts of the program? = Yes

PS5a_PW How often did your **work** keep you from completing some parts of the program?

- Never (1)
- Rarely (2)
- Sometimes (3)
- Often (4)

PS6_PW Have **family activities**, like caring for children or other family members, kept you from completing some parts of the program?

- Yes (1)
- No (0)

Display This Question:

If Have family activities, like caring for children or other family members, kept you from completin... = Yes

PS6a_PW How often did **family activities** keep you from completing some parts of the program?

- Never (1)
- Rarely (2)
- Sometimes (3)
- Often (4)

PS7_PW Has the **cost of food** kept you from completing some parts of the program?

- Yes (1)
- No (0)

Display This Question:

If Has the cost of food kept you from completing some parts of the program? = Yes

PS7a_PW How often did the **cost of food** keep you from completing some parts of the program?

- Never (1)
- Rarely (2)
- Sometimes (3)
- Often (4)

PS8_PW Have **community activities** (e.g., volunteering, church) kept you from completing some parts of the program?

- Yes (1)
- No (0)

Display This Question:

If Have community activities (e.g., volunteering, church) kept you from completing some parts of the... = Yes

PS8a_PW How often did **community activities** keep you from completing some parts of the program?

- Never (1)
- Rarely (2)
- Sometimes (3)
- Often (4)

| Page Break |  |
| --- | --- |

PS9_PW Do you think that the time commitment involved in the following aspects of the program was **too much, just right, or not enough**?

|  | Too much (1) | Just right (2) | Not enough (3) |
| --- | --- | --- | --- |
| Amount of time spent planning meals. (PS9a_PW) |  |  |  |
| Amount of time spent preparing and cooking meals. (PS9b_PW) |  |  |  |
| Amount of time spent tracking food on the app. (PS9c_PW) |  |  |  |
| Amount of time spent engaging in physical activity. (PS9d_PW) |  |  |  |
| Amount of time spent tracking physical activity on the app. (PS9e_PW) |  |  |  |
| Amount of time watching incentaHEALTH videos (PS9f_PW) |  |  |  |
| Amount of time spent consulting with online coaches (PS9g_PW) |  |  |  |
| Amount of time spent consulting with health professionals (community health workers/community health nurses, etc.) (PS9h_PW) |  |  |  |
