# Supplementary material for: Community-engaged design of a virtual diabetes prevention program intervention with care coordination
Source: BMC Health Serv Res. 2026 Jun 13;26:1166. doi: 10.1186/s12913-026-14860-0 (PMC13491910; doi:10.1186/s12913-026-14860-0)
Supplement: Supplementary file 12 [file 12913_2026_14860_MOESM9_ESM.docx]

Additional File

Technology Survey Results

Table S1: Technology Survey Responses among Ansonia, Derby, and New Haven Residents

(N = 19)

| **Question** | **N (%)** |
| --- | --- |
| **City/Town** | |
| Ansonia | 3 (15.8) |
| Derby | 4 (21.1) |
| New Haven | 9 (47.4) |
| Shelton | 1 (5.3) |
| Seymour | 2 (10.5) |
| **Internet access in home** | 16 (84.2) |
| **If Internet access only outside of home** | |
| Work | 2 (10.5) |
| Family or friend’s home | - |
| Library or public space | - |
| **Lives with someone who can help access Internet** | 8 (42.1) |
| **Type of Internet access in home** | |
| Wifi | 16 (84.2) |
| Limited phone data plan | - |
| Unlimited phone data plan | - |
| Not sure | - |
| Other | - |
| **Frequency of Internet access** | |
| Several times a day | 16 (84.2) |
| Once or twice a day | - |
| Few times a week | 3 (15.8) |
| Once a week | - |
| Less than once a week | - |
| Never | - |
| **Comfort level with independent Internet usage** | |
| Very comfortable | 14 (73.7) |
| Somewhat comfortable | 4 (21.0) |
| Not very comfortable | 1 (5.3) |
| I do not use Internet | - |
| **Used computer or smartphone to track diet or exercise** | |
| Yes, currently uses | 1 (5.3) |
| Yes, no longer uses | 5 (26.3) |
| No | 13 (68.4) |

Figure S1

How often do you use each of the following devices to access the Internet?

|  | Always | Often | Sometimes | Never |
| --- | --- | --- | --- | --- |
| Other | 0 | 0 | 2 | 6 |
| Other type of mobile phone | 1 | 1 | 1 | 8 |
| A smartphone or tablet owned by a friend or family member | 0 | 0 | 4 | 14 |
| A computer at a friend's or family member's home | 0 | 0 | 6 | 12 |
| A computer in a shared or public space | 0 | 0 | 6 | 12 |
| A smartphone or tablet that I own | 9 | 4 | 3 | 1 |
| A computer that I own | 9 | 3 | 5 | 3 |

Table S2: Frequency of device use to access the Internet (N = 19)

|  | **Always**  N (%) | **Often**  N (%) | **Sometimes**  N (%) | **Never**  N (%) | **No Response**  N (%) |
| --- | --- | --- | --- | --- | --- |
| **Computer** | 9 (47.3) | 3 (15.8) | 4 (21.1) | 3 (15.8) | 0 (0.0) |
| **Smartphone** | 9 (47.3) | 4 (21.1) | 3 (15.8) | 1 (5.3) | 2 (10.5) |
| **Public computer** | 0 (0.0) | 0 (0.0) | 6 (31.6) | 12 (63.2) | 1 (5.3) |
| **Computer friend/family** | 0 (0.0) | 0 (0.0) | 6 (31.6) | 12 (63.2) | 1 (5.3) |
| **Smartphone friend/family** | 0 (0.0) | 0 (0.0) | 4 (21.1) | 14 (73.7) | 1 (5.3) |
| **Other mobile** | 1 (5.3) | 1 (5.3) | 1 (5.3) | 8 (42.1) | 8 (42.1) |
| **Other** | 0 (0.0) | 0 (0.0) | 2 (10.5) | 6 (31.6) | 11 (57.9) |
