# Supplementary material for: Community-engaged design of a virtual diabetes prevention program intervention with care coordination
Source: BMC Health Serv Res. 2026 Jun 13;26:1166. doi: 10.1186/s12913-026-14860-0 (PMC13491910; doi:10.1186/s12913-026-14860-0)
Supplement: Supplementary file 13 [file 12913_2026_14860_MOESM6_ESM.docx]

Additional File 6
